# Understanding Physical Activity Determinants in an HIV Self-Management Intervention: Qualitative Analysis Guided by the Theory of Planned Behavior

**DOI:** 10.2196/47666

**Published:** 2023-09-14

**Authors:** Gabriella Sanabria, Brady Bushover, Sarah Ashrafnia, Evette Cordoba, Rebecca Schnall

**Affiliations:** 1 College of Public Health University of South Florida Tampa, FL United States; 2 Columbia University Mailman School of Public Health New York, NY United States; 3 Division of Scholarship and Research Columbia University School of Nursing New York, NY United States

**Keywords:** HIV/AIDS, mobile Health, mHealth, fitness tracker, physical activity, self-management, HIV, AIDS, activity, mortality, app, fitness, qualitative, tracking, behavior, mobile phone

## Abstract

**Background:**

People living with HIV have long life expectancy and are experiencing more comorbid conditions, being at an increased risk for developing cardiovascular disease (CVD) and diabetes, further exacerbated due to the HIV or inflammatory process. One effective intervention shown to decrease mortality and improve health outcomes related to CVD and diabetes in people living with HIV is increased regular physical activity. However, people living with HIV often fall short of the daily recommended physical activity levels. While studies show that mobile health (mHealth) can potentially help improve people’s daily activity levels and reduce mortality rates due to comorbid conditions, these studies do not specifically focus on people living with HIV. As such, it is essential to understand how mHealth interventions, such as wearables, can improve the physical activity of people living with HIV.

**Objective:**

This study aimed to understand participants’ experiences wearing a fitness tracker and an app to improve their physical activity.

**Methods:**

In total, 6 focus groups were conducted with participants who completed the control arm of a 6-month randomized controlled trial (ClinicalTrials.gov NCT03205982). The control arm received daily walk step reminders to walk at least 5000 steps per day and focused on the overall wellness of the individual. The analysis of the qualitative focus groups used inductive content analysis using the theory of planned behavior as a framework to guide and organize the analysis.

**Results:**

In total, 41 people living with HIV participated in the focus groups. The majority (n=26, 63%) of participants reported their race as Black or African American, and 32% (n=13) of them identified their ethnicity as Hispanic or Latino. In total, 9 major themes were identified and organized following the theory of planned behavior constructs. Overall, 2 major themes (positive attitude toward tracking steps and tracking steps is motivating) related to attitudes toward the behavior, 2 major themes (social support or motivation from the fitness tracker and app and encouragement from family and friends) related to participant’s subjective norms, 1 theme (you can adjust your daily habits with time) related to perceived behavioral control, 2 themes (reach their step goal and have a healthier lifestyle) related to participant’s intention, and 2 themes (continuing to walk actively and regularly wearing the fitness tracker) related to participant’s changed behavior. Participants highlighted how the mHealth interface with the avatar and daily step tracking motivated them to both begin and continue to engage in physical activity by adjusting their daily routines.

**Conclusions:**

Findings from this study illustrate how features of mHealth apps may motivate people living with HIV to start and continue sustained engagement in physical activities. This sustained increase in physical activity is crucial for reducing the risk of comorbid conditions such as diabetes or CVD.

**Trial Registration:**

ClinicalTrials.gov NCT03205982; https://classic.clinicaltrials.gov/ct2/show/NCT03205982

## Introduction

People living with HIV are living longer lives due to earlier detection and advanced treatment [1,2]. As part of living longer, people living with HIV are experiencing more comorbid conditions and are at increased risk for developing cardiovascular disease (CVD) and diabetes that are further exacerbated because of the HIV or inflammatory process, facing a growing prevalence of mortality associated with these comorbid conditions [3-5]. One effective intervention shown to decrease mortality and improve health outcomes related to CVD and diabetes in people living with HIV is increased regular physical activity [1,6]. Increasing physical activity is critical because of the comorbidities associated with CVD and diabetes [1,7]. However, physical activity rates among people living with HIV remain low compared to the general population [1,7,8]. In a systematic review, about half of the people living with HIV reached the recommended 150 minutes of physical activity per week and, on average, fell short of walking the recommended 10,000 daily steps [7]. There is a need to develop effective strategies to increase physical activity among people living with HIV [1,7].

One such strategy to increase physical activity among people living with HIV is the use of mobile health (mHealth) technologies. mHealth technology uses tools that allow individuals to wirelessly and remotely access health information to manage and maintain one’s health [9,10]. Such devices may include mobile apps on smartphones or fitness trackers [11]. Recent studies have demonstrated that forms of mHealth technology, including wearable devices with goals and challenges, can facilitate improvements in physical activity [12-16]. Using mobile technology offers ease and efficiency in tracking activities among patients due to its instant feedback and support while being cost-effective [11,14,17]. However, none of these studies have been conducted specifically for people living with HIV. As such, this study aimed to understand the experiences of people living with HIV wearing a fitness tracker in conjunction with the HealthStar app interface (WiseApp) on their physical activity using the theory of planned behavior (TPB) to help guide the analysis.

## Methods

### Sampling and Recruitment

This study was conducted following participants’ completion of a 6-month randomized controlled trial (RCT) with 200 people living with HIV in New York (100 randomly assigned to the intervention and 100 randomly assigned to the control). Full details of the trial can be found on ClinicalTrials.gov (NCT03205982), and the results of the study and study protocol have been published elsewhere [18,19]. In short, the goal of the study was to improve medication adherence in people living with HIV. Participants in the RCT were randomized to one of two arms: (1) an intervention arm, in which the WiseApp interface provided daily medication reminders, videos surrounding living with HIV and taking medication, health surveys, and a chat room, or (2) a control arm, in which the WiseApp interface provided daily walk step reminders, videos focusing on overall wellness including diet, sleep, and exercise, health surveys, and a chat room [19]. Following the 6-month trial, research staff conducted follow-up focus groups with a subset of study participants from the control arm who received daily reminders to walk at least 5000 steps per day and whose focus was on overall wellness.

### Ethics Approval

The institutional review board of Columbia University Medical Center reviewed and approved all research activities (protocol AAAQ9957). Study activities took place at the Columbia University School of Nursing. A research coordinator explained the procedures to the study participants, and written documentation of informed consent was obtained prior to the commencement of study activities. Participants were given US $30 gift cards as compensation for their time.

### Procedures

Using an open-ended, semistructured focus group guide (Textbox 1), a moderator (research staff or faculty at Columbia University School of Nursing) facilitated the focus group discussion, and participants were encouraged to express their experiences, perception, satisfaction, and impact of using the fitness tracker linked to the WiseApp while in the trial. All focus group sessions were audio-recorded, and data collection continued until saturation was reached [20]. Audio recordings from the focus groups were transcribed verbatim.

**Focus group guide to assess experience using the fitness tracker and WiseApp.**

**Experience using the fitness tracker (Fitbit device or watch and Fitbit app)**

- What are some of the things that you like about using the fitness tracker syncing to the HealthStar app?
- Please describe your experience using the fitness tracker in your daily life.
  Probe: Do you have it with you all the time or sometimes? When or where did you take it off? Why?
- How easy or difficult was it for you to use the fitness tracker?
  Probe: Would you be confident about setting up the Fitbit app for the first time without assistance? (setup of Fitbit app, Fitbit device or watch, and then linking to HealthStar app)
- Please describe your experience dealing with any difficulties with the fitness tracker.
  Probe: (Technical issues) Not working or syncing? Problems with band or charger? Have you ever lost or stolen the Fitbit? Where and how?
- What are your thoughts about the design and function of the fitness tracker?
  Probe: Was it comfortable to wear? Did it fall off easily? Do you think it accurately monitored your steps?
- What have you used in the past for tracking your physical activity (eg, walk steps) and receiving reminders about your daily walk step goal? How does this compare?
- Please describe your experience monitoring your daily physical activity.
  Probe: The number of daily steps and how often you completed your daily step goal?
- Please describe your experience receiving reminders about completing your step goal.
  Probe: Reminders sent on the HealthStar app? Did you like the reminders to complete your walk steps? Do you think the reminders motivated you to complete the steps? Why or why not?

**Impact of the fitness tracker (Fitbit device or watch and Fitbit app)**

- How do you think that your physical activity (ie, daily step goal completion) changed after using the fitness tracker for 6 months?
- How did the fitness tracker change your physical activity?
- How did the HealthStar app help you improve your physical activity?
- What are some of the ways that your physical activity may change through the use of the fitness tracker?
- What are some of the ways that your physical activity may change through the use of the HealthStar linked with the fitness tracker?
- How confident are you in your ability to keep up with your physical activity in everyday life?
- Please describe how the fitness tracker did or can change your quality of life.
  Probe: Have you noticed changes in various aspects of your life? More aware of your physical activity, general health, etc?
- At the completion of this study, would you want to keep using the fitness tracker or HealthStar app? Why or why not?

### Data Analysis

**Figure 1:**
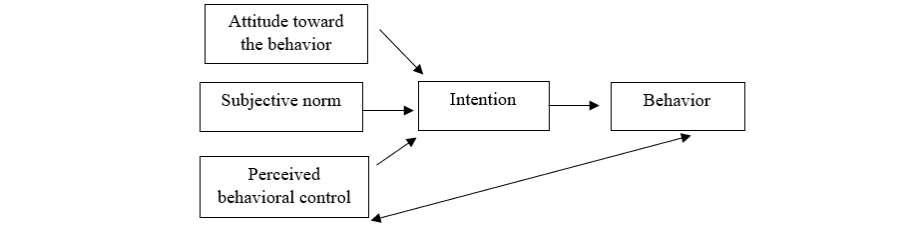
Theory of planned behavior model.

Data analysis was guided by the TPB [21]. TPB was chosen, as previous studies used the TPB to help predict and explain determinants of physical activity in individuals [22,23]. The TPB is a modified extension of the theory of reasoned action and includes the concepts of perceived behavioral control and how an individual's perceived control can lead to behavior change [24]. The TPB explores the relationship between how an individual’s beliefs, attitudes, and intentions impact their behavior and if a behavior change occurs (Figure 1) [21,24]. Based on the TPB, an individual’s intention is the largest determinant if a behavior occurs, and one’s intention, or the likelihood that an individual will perform the behavior, is influenced by an individual’s attitude, subjective norm, and perceived behavioral control [21,24]. The concept of attitude toward the behavior refers to a person’s evaluation of the behavior, subjective norm refers to the normative beliefs that result in perceived social pressure, and perceived behavioral control refers to the beliefs that one can exercise control over performing a behavior [21,24].

Inductive content analysis was used to code the transcripts in Microsoft Word (Microsoft Corp) [25,26]. The codebook was created using Microsoft Excel (Microsoft Corp). Microsoft Excel was used to manage the data analysis as text experts from transcripts were entered under the themes that emerged. Research team members (GS, BB, and SA) independently reviewed and coded the focus groups’ transcripts. GS noted any discrepancies between the codes from each team member (GS, BB, and SA). Research team members (GS, BB, and SA) discussed the discrepancies until a consensus was reached. The TPB framework was used to guide the coding and thematic analysis, noting patterns across the focus group data.

## Results

### Sample

A total of 41 out of the 100 people who completed the control arm of the RCT participated in the 6 focus group sessions. Convenience sampling was used by calling participants who completed the 6-month trial to participate in the focus groups [27]. The only inclusion criterion was that the participant completed the 6-month RCT prior to participating in the focus group. Each focus group had between 4 and 10 (average 6.8) participants in the sessions. The mean age of study participants was 50 (SD 10.1; range 27-65) years. The majority (n=26, 63%) of participants reported their race as Black or African American, and 32% (n=13) identified as Hispanic or Latino. Of the participants, 63% (n=26) of them were assigned male sex at birth, and 49% (n=20) of participants identified their marital status as single. Approximately, 44% (n=18) of them reported their annual income as less than US $10,000. About 42% (n=17) of them reported their highest education level as some college, and 84% (n=32) had Medicaid or Medicare as their health insurance provider.

### Major Themes

#### Overview

We identified 9 major themes ex post facto, which were organized following the TPB constructs. There were 2 major themes relating to attitudes toward the behavior, 2 major themes relating to the participant’s subjective norms, 1 theme relating to perceived behavioral control, 2 themes relating to the participant’s intention, and 2 themes relating to the participant’s changed behavior [21,24]. Table 1 provides sample quotes for each of the TPB constructs and their related themes.

**Table 1:** Themes and example quotes of the theory of planned behavior constructs.

Construct and theme		Sample quote
**Attitude toward the behavior**		
	Positive attitude toward tracking steps	“Oh, I liked the fitness tracker because of the steps, I can keep track of my calories and various other things that the app offers. And, I found it was very beneficial to me. It was new, it was a new thing, and I enjoyed it. I found it very beneficial for me.” [Focus group 4]
	Tracking steps is motivating	“Seeing the end of the week results were motivators…” [Focus group 1]
**Subjective norms**		
	Social support or motivation from the fitness tracker and app	“Having a Fitbit is like having your right-hand person. It monitors you. It tells you when you’re doing good; you’re not doing it…It keeps me on point on everything…what I have to do next. It keeps me alert. That’s what’s good about the Fitbit.” [Focus group 6]
	Encouragement from family and friends	“What I did like was that because my father had a Fitbit and his wife had a Fitbit, we would challenge each other.” [Focus group 1]
**Perceived behavioral control**		
	You can adjust your daily habits with time	“And instead of me driving all the time, I walk, so I can get my little exercise.” [Focus group 2]
**Intention**		
	Reach their step goal	“I want to get that 10,000 mark in, and watch it go off, and watch the little stars go off and all of that.” [Focus group 2]
	Have a healthier lifestyle	“It [the fitness tracker] has helped me by far, my health, and my motivation to help myself.” [Focus group 5]
**Behavior**		
	Continuing to walk actively	“I'm doing more now because when I got this on I like to complete what I’ve started. So now when I wake up in the morning, I like to start this and at least get the 10,000 steps in by the end of the day. And, I have done it every day so far.” [Focus group 5]
	Regularly wearing the fitness tracker	“My Fitbit is a part of me now. I wake up and put it on every morning, like I would do my earrings.” [Focus group 1]

#### Attitudes Toward the Behavior

Concerning an individual’s behavioral belief produces favorable or unfavorable attitudes toward the behavior. Two recurring themes emerged from the inductive analysis: “positive attitude toward tracking steps” and “tracking steps is motivating.”

##### Positive Attitude Toward Tracking Steps

Almost all participants in the focus groups had a positive attitude toward using the fitness tracker and found it very beneficial for their daily life. One participant highlighted how instructive the app was stating,

This was very educational. It was good. It was beneficial.

Focus group 1

Participants in general found the fitness tracker and its step count very helpful as one participant described,

Oh, I liked the fitness tracker because of the steps, I can keep track of my calories and various other things that the app offers. And, I found it was very beneficial to me. It was new, it was a new thing, and I enjoyed it. I found it very beneficial for me.

Focus group 4

While the majority of individuals had favorable attitudes toward step tracking, it is important to note that a few participants did not enjoy tracking their steps as an activity or did not like the Fitbit itself as a wearable. One participant stated,

I hate that little skinny thing. I swear to god, it came out just like that. I got frustrated for a while too because I really didn’t want to do it. Then it reminded me to do it. And I'm like, I'm taking this thing off.

Focus group 4

##### Tracking Steps Is Motivating

Most participants also particularly enjoyed tracking their steps and viewed the daily step goal as motivation to continue working toward reaching their step goal and staying active. Several participants stated,

Seeing the end of the week results were motivators…

Focus group 1

I would look in the app. I would do more than 5000. And I'm like…yay!

Focus group 2

Like if you have a goal of 10,000 steps, and then at the end you see you’ve done like over 10,000, you're very proud of that.

Focus group 3

#### Subjective Norms

There were 2 normative beliefs that resulted in perceived social pressure on the participants and impacted the individual’s intentions. The themes under subjective norms were “social support or motivation from the fitness tracker and app” and “encouragement from family and friends.”

##### Social Support or Motivation From the Fitness Tracker and App

Participants felt that the fitness tracker linked to the WiseApp provided motivation and support to improve their physical activity and reach their step goals. Participants described,

It made everybody kind of, anybody that got into it, then you like the idea of getting appreciated for the work you’ve done.

Focus group 2

Having a Fitbit is like having your right-hand person. It monitors you. It tells you when you’re doing good; you’re not doing it…It keeps me on point on everything…what I have to do next. It keeps me alert. That’s what’s good about the Fitbit.

Focus group 6

Participants also felt that in particular, the WiseApp avatar, the turtle, was also motivating with how it responded to participants reaching their daily goal stating,

I just liked the cute little turtle. I would log on to the app just to see the little happy turtle. The turtle was happy I got my 5,000 steps in.

Focus group 1

Yes, the turtle reminds me of where I am at, where I am supposed to be, where am I going, what am I doing, how many steps am I walking… It keeps me focused.

Focus group 6

##### Encouragement From Family and Friends

In the focus group, another prominent factor discussed as impacting an individual’s intention to make a behavior change was motivation and support from others. The comments and encouragement from family and friends motivated participants to continue moving forward with their step goal. Some participants found the comments from others encouraging as one participant mentioned,

It’s okay because I am looking at myself and saying, hey, you really did tone down. I don’t see it, but other people see it.”

Focus group 1

Others found comparing the Fitbit walk steps with those of their family and friends both motivating and encouraging as several people described,

What I did like was that because my father had a Fitbit and his wife had a Fitbit, we would challenge each other.

Focus group 1

Yeah. You compare. Then you're talking. I used to walk all the time when I was with a whole bunch of girls. We’d get together.

Focus group 3

We compete against each other, though not in the same place, you know, so it’s really, really good. It’s good for our kids and our grandkids and bring them into what we do, you know.

Focus group 5

#### Perceived Behavioral Control

Within the perceived behavioral control component, which focused on the level of an individual’s belief that they have power or can exercise control over performing a behavior, the resounding theme was “you can adjust your daily habits with time.”

##### You Can Adjust Your Daily Habits With Time

Participants felt that they had the power to increase their physical activity levels by changing their daily habits to meet their step goals. Participants mentioned several ways in which they adjusted their routines,

Instead of catching the bus down two blocks then I’ll just go ahead and walk trying to get my steps in.

Focus group 1

And instead of me driving all the time, I walk, so I can get my little exercise.

Focus group 2

I go check my mailbox three or four times on Sunday, just to take the stairs.

Focus group 2

While participants felt that it was possible to adjust their daily habits, some individuals felt that they needed longer than their 6-month duration in the study to continue with the habit. This can be seen as one participant commented,

Because I feel like a year is a good enough time to develop a habit. Because after six months, that’s about breaking a habit. But to develop a habit, a good habit, I would say you need more time.

Focus group 3

#### Intention

According to the TPB, the attitudes, subjective norms, and perceived behavioral control, all impact the participants’ intention to perform a behavior [21]. In analyzing the focus groups, 2 intentions emerged from the participants regarding their experience using fitness tracker linked to the WiseApp: “reach their step goal” and “have a healthier lifestyle.”

##### Reach Their Step Goal

Participants discussed their main intention to reach their step goal on the WiseApp. Many participants mentioned how they strived to reach their daily step target stating,

It was basically like monitoring my steps and putting them all together and being able to see, okay, this is how much I did. Maybe I should do a little more, like this day or that day. And planning, you know, scheduling workouts for use in the Fitbit.

Focus group 5

So between being off the bike and walking around, if I could do 10,000 steps a day, that was like a target.

Focus group 2

I want to get that 10,000 mark in, and watch it go off, and watch the little stars go off and all of that.

Focus group 2

##### Have a Healthier Lifestyle

In addition to meeting their step goal on the WiseApp, participants also mentioned a desire to stay healthy as one of their key intentions driving their engagement with the fitness tracker. Participants stated that the fitness tracker created an awareness of a healthy lifestyle, for example,

You know, I just started using it like every now and then. I think what they gave me was awareness to how many steps to a more healthy lifestyle. That is what it really did; it gave me awareness to it.

Focus group 5

Participants also discussed how the fitness tracker created momentum and motivation to continue prioritizing their health stating,

I was going to say that like before this study, I was 30-pounds heavier. So, I lost that. And along with the pandemic, and that whole thing, you know, the Fitbit was instrumental in that experience. But like afterwards, I'm still like, you know, the momentum is still carrying me, and you know, I'm still rolling with it, and now it’s automatic.

Focus group 5

It [the fitness tracker] has helped me by far, my health, and my motivation to help myself.

Focus group 5

#### Behavior

Finally, the participants’ intentions led to behavior changes after using the fitness tracker linked to the WiseApp during the trial. The 2 major behavior changes that occurred were “continuing to walk actively” and “regularly wearing the fitness tracker.”

##### Continuing to Walk Actively

Participants mentioned that they continue to focus on actively walking and increasing their physical activity after participating in the study, trying to meet the step goals that they set for themselves on the Fitbit app. Several participants described their current walking activities,

It’s been such a motivator for me to be focused on 10,000 steps. I don’t do it every day, but at least five times a week.

Focus group 1

Yeah, I became more active, and still be active.

Focus group 3

I'm doing more now because when I got this on I like to complete what I’ve started. So now when I wake up in the morning, I like to start this and at least get the 10,000 steps in by the end of the day. And, I have done it every day so far.

Focus group 5

I’m still walking the same thing, you know…I'm always walking around. I like to keep tabs on it.

Focus group 3

##### Regularly Wearing the Fitness Tracker

The fitness tracker also became a consistent accessory for participants’ wardrobes. Various participants described regularly wearing the tracker and actively engaging with it. Several participants highlighted their dependence on the fitness tracker stating,

If I don’t have it, I feel like I'm missing something. I only take it off to shower.

Focus group 3

It was easy. I knew where to put it. And I knew where it was at when I get up in the morning. The first thing I do, wash my face, brush my teeth, and get my Fitbit on.

Focus group 3

My Fitbit is a part of me now. I wake up and put it on every morning, like I would do my earrings.

Focus group 1

## Discussion

### Principal Findings

Studies show that mobile apps can help enable behavior change and improve health outcomes in people with chronic disease and people living with HIV [28-31]. People living with HIV now experience an increasing burden of chronic illness and have higher morbidity and mortality rates for non–AIDS-related health complications, like CVD, than the general population [1,3,7,32]. Physical activity has been shown to have various health benefits such as reducing CVD risk, increasing physical function and vitality, and improving an individual's overall cardiometabolic profile [8]. Understanding physical activity levels and the motivation for physical activity among people living with HIV is key to improving long-term health outcomes like cardiovascular and metabolic health [1,7,33]. An important strength of the study is that while the TPB has been applied to predicting behavioral intentions and changes in physical activity, this is the first time TPB is applied to people living with HIV to understand their determinants of physical activity and engagement with an mHealth intervention [23,34].

When analyzing the focus groups using the TPB framework, participants in the study exhibited high levels of behavioral control. Many participants believed that they could adjust their daily habits to meet their physical activity goals provided by the WiseApp interface. This perceived behavioral control is vital, as it impacts both an individual’s intention and whether or not they perform the desired behavior of reaching physical activity goals [21]. Based on these findings, we believe that it is not that individuals feel they cannot change their behavior to increase physical activity or that the behavior is outside of their control. Rather, there needs to be continuous motivation to encourage participants to be physically active, such as the avatar and walk step reminders in the WiseApp for a prolonged period of time.

While this research shows that daily reminders help keep people engaged in physical activity, our focus groups added to the literature by showing that it is not just reminders but reminders in conjunction with an app’s avatar that helps to create a strong motivation to continue engaging in physical activity [16,35]. Our study results also align with prior research that shows that mHealth apps and engagement with app interfaces can promote continued engagement and motivation in physical activity and wellness goals [36,37]. However, our study adds to the literature as the prior studies were not centered on people living with HIV.

Finally, it is important to note that the participants in the focus groups had 2 central intentions that were driving their behaviors. The first intention was to reach the daily step goals, and the second was to live a healthier lifestyle. Following the TPB framework, the participants’ behavioral intentions led them to the behavior changes of actively walking and regularly wearing fitness trackers. Findings from this study suggest that a fitness tracker linked to an app interface may help people living with HIV actively engage in behavior changes and increase their activity levels. The fitness tracker and app with avatar combination provided both awareness and motivation for participants to better their health by improving their physical activity by adjusting their daily routines.

Future research should continue to expand upon this work of understanding how mHealth technology interfaces can be combined and integrated to motivate and sustain the increase in physical activity levels in people living with HIV. As this intervention included both a fitness tracker and an app that had multiple features including the avatar, videos focusing on overall wellness, health surveys, and a chat room, it would be important to know what aspects were most effective at motivating and assisting with sustained physical activity levels and improving the overall health and well-being of people living with HIV. For example, as participants mentioned the avatar was motivational, perhaps this was the key feature of the app that helped with physical activity the most, and the educational videos were not as effective. Understanding what combination of wearable and app components is most effective to improve sustained physical activity levels and the overall well-being of people living with HIV would be the next step in future investigations.

### Limitations

There are some limitations of this study to note. Study participants may have been more likely to engage in physical activity and behavior changes as this study focused on improving the overall quality of life for people living with HIV. Those who agreed to participate in the focus groups may have been those who were most influenced by the fitness tracker experience. Additionally, the participants were predominantly Black or African American males who did not identify as Hispanic or Latino, made less than US $10,000 per year, and lived in New York City. Feedback provided by the focus group members may differ for other groups of individuals, including females, people of different races, ethnicities, and income levels, hindering the generalizability of the results. Finally, we were also unable to validate the self-report fitness tracker discussions with quantitative data regarding daily walk steps and app use. Despite these limitations, we are able to begin to understand the interaction between mHealth apps and wearable technologies (fitness trackers) and their influence on improving physical activity levels among people living with HIV. Improving physical activity can lead to reducing their risk of CVD and reduce the morbidity and mortality that this community experiences related to CVD.

### Conclusions

This paper uses a TPB framework to understand what factors are related to increasing physical activity and the impact of using a fitness tracker in conjunction with an mHealth app for people living with HIV. Participants highlighted how the mHealth interface with the avatar and daily step tracking was a motivating feature to help them start and continue to engage in physical activity, helping them to meet their daily goals. Participants became more involved with their physical activity after wearing the fitness tracker during the study. Some participants continued to wear their fitness tracker and track their steps even after study completion. Further research should be conducted to understand how avatars can promote prolonged engagement in physical activity in order to enhance and promote better cardiovascular function in a more diverse sample of people living with HIV.

## Abbreviations

CVD: cardiovascular disease
mHealth: mobile health
RCT: randomized controlled trial
TPB: theory of planned behavior

## Data Availability

Data sharing is not applicable to this paper as no quantitative data sets were generated or analyzed during this study.
